# Emerging function of mTORC2 as a core regulator in glioblastoma: metabolic reprogramming and drug resistance

**DOI:** 10.7497/j.issn.2095-3941.2014.04.004

**Published:** 2014-12

**Authors:** Si-Han Wu, Jun-Feng Bi, Timothy Cloughesy, Webster K. Cavenee, Paul S. Mischel

**Affiliations:** ^1^Ludwig Institute for Cancer Research, University of California, San Diego, La Jolla, CA 92093, USA; ^2^Neuro-Oncology Program, University of California, Los Angeles, CA 90095, USA

**Keywords:** Glioblastoma, mTOR, metabolic reprogramming, mTORC2, Warburg effect, PI3K

## Abstract

Glioblastoma (GBM) is one of the most lethal human cancers. Genomic analyses define the molecular architecture of GBM and highlight a central function for mechanistic target of rapamycin (mTOR) signaling. mTOR kinase exists in two multi-protein complexes, namely, mTORC1 and mTORC2. These complexes differ in terms of function, regulation and rapamycin sensitivity. mTORC1 is well established as a cancer drug target, whereas the functions of mTORC2 in cancer, including GBM, remains poorly understood. This study reviews the recent findings that demonstrate a central function of mTORC2 in regulating tumor growth, metabolic reprogramming, and targeted therapy resistance in GBM, which makes mTORC2 as a critical GBM drug target.

## Introduction

Glioblastoma (GBM) is the most common malignant primary brain cancer of adults, accounting for more than 45% of malignant primary brain and CNS tumors. GBM is also one of the most lethal cancers, with a median survival of only 15 months for patients despite combined treatment, including surgery, radiotherapy, and chemotherapy^1^.

GBM was one of the first cancer types profiled by The Cancer Genome Atlas project and is now one of the most genomically well-characterized forms of human cancer for which a central function of dysregulated growth factor receptor signaling has been demonstrated^2^^,^^3^. Receptor tyrosine kinase gene amplification and mutations, PI3K catalytic and regulatory subunit genetic mutations, and PTEN gene deletion and mutation all result in constitutive PI3K pathway activation in the majority of GBMs, thus rendering the downstream effect or mechanistic target of rapamycin (mTOR) a compelling GBM drug target^4^.

mTOR is a serine threonine kinase that exists in two distinct complexes, mTOR complexes I and II (mTORC1 and mTORC2), which differ in terms of their regulation, function, and responsiveness to the allosteric inhibitor, rapamycin. mTORC1, which contains mTOR kinase in complex with six known components including Raptor, links upstream growth factor receptor signaling to downstream protein translation and cell proliferation through PI3K^5^. mTORC1 integrates growth factor receptor signaling into amino acid and energy status to ensure sufficient nutrients and ATP to enable tumor proliferation and cell growth^6^. mTORC1 also regulates protein degradation^7^, ribosome biogenesis^8^, glucose, lipid and nucleotide metabolism^5^^,^^9^^,^^10^, as well as autophagy^11^. In contrast to that of mTORC1, the function of mTORC2 remains poorly understood. This study reviews mTOR signaling in GBM with a focus on the newly identified central function of mTORC2 in GBM pathogenesis.

## mTORC2 *vs*. mTORC1: structure and signaling

mTORC1 and mTORC2 differ in terms of subcellular localization, regulation, function, and sensitivity to the allosteric inhibitor, rapamycin^12^. Structurally, both share four common components: mTOR, mLST8, Deptor, and Tti1/Tel2^9^. In addition to the common components, mTORC1 contains two other proteins PRAS40 and Raptor, whereas mTORC2 has three other proteins, Rictor, mSIN1, and Protor-1^9^.

mTORC1 signaling is activated in response to the growth factor receptor signaling and nutrient availability through complementary and independent mechanisms^6^. This complex is activated in the majority of adult GBMs, partly as a consequence of growth factor receptor signaling through the PI3K-AKT pathway^13^. TSC1/2 is the key regulator of mTORC1 and inhibits mTORC1 signaling by converting the mTORC1 activator Rheb to its inactive state^14^^,^^15^. Growth factors stimulate PI3K/AKT activity, thereby inhibiting the tumor suppressors TSC1/2, thus resulting in mTORC1 activation^13^. AKT also activates mTORC1 by phosphorylating PRAS40 and dissociating PRAS40 from mTORC1^9^. mTORC1 signaling is regulated by the nutrient and energy levels. Amino acids act independently of TSC1/2 to activate mTORC1 through Rag GTPases by promoting the translocation of mTORC1 to the lysosomal surface^16^^,^^17^. The energy sensor, AMPK, also regulates mTORC1 by phosphorylating Raptor to inhibit mTORC1 activity^18^.

Phosphatidic acid is required for the stability and activity of mTORC1^19^. Thus, mTORC1 integrates growth factors, energy status, and amino acids to regulate numerous cellular processes, including protein translation, lipid synthesis, energy metabolism, lysosome biogenesis, and autophagy to promote tumor proliferation and cell growth. mTORC1 promotes protein translation by phosphorylating S6K1 and 4E-BP1^12^, as well as controls proteasome-mediated protein degradation through an NRF1-dependent mechanism, thus balancing cellular protein synthesis and degradation^7^. More importantly, mTORC1 is a key negative regulator of PI3K signaling that serves as a homeostatic rheostat. However, mTORC1, as a negative feedback loop, inhibits PI3K signaling by directly or S6K1-dependently phosphorylating IRS1 and promoting IRS1 degradation^20^^,^^21^.

In contrast to those of mTORC1, mTORC2 regulation and function, particularly in cancer, remain poorly understood. A recent work suggests that growth factor signaling through PI3K promotes mTORC2-ribosome binding to stimulate TORC2 kinase activity^22^. In GBM, EGFRvIII and PTEN loss, both of which can potentially promote PI3K signaling, also stimulate mTORC2 kinase^23^. PI3K-independent mechanisms of mTORC2 activation, including WNT-LRP5 signaling through the small GTPase, RAC1, during osteoblast differentiation^24^, miR-29 regulation of YAP and Hippo pathway activation^25^, and Notch signaling^26^^,^^27^, have recently been described.

mTORC1 and mTORC2 are reciprocally regulated. Downstream of mTORC1, S6K1 inhibits mTORC2-dependent phosphorylation of AKT by phosphorylating Rictor on Thr1135^28^ and mSIN1 on Thr86 and Thr398^29^. Until relatively recently, the majority of mTORC2 activity was believed to be mediated by its phosphorylation of AKT on Ser473, as well as by controlling the folding and stability of AKT protein, thus promoting maximal AKT signaling^30^^-^^33^. Accordingly, mTORC2 can promote mTORC1 signaling in an AKT-dependent manner. However, mTORC2 activates the additional members of the AGC subfamily of kinases, including SGK1^34^ and PKC-α^35^, to regulate cell survival, metabolism, and cytoskeletal organization. Thus, a Drosophila model of EGFR-PI3K-driven gliomas identified mTORC2 as a requirement for tumor formation, independent of AKT and mTORC1^36^. mTORC2 promotes GBM growth, survival, and chemotherapy resistance through SGK1-dependent and NF-κB-dependent signaling^23^^,^^37^^,^^38^. Therefore, both mTORC1 and mTORC2 should be inhibited to prevent GBM tumor growth.

The mTOR inhibitor rapamycin interacts with the FKBP12 protein and then binds to the FRB domain in mTOR to inhibit its kinase activity allosterically^39^. mTORC1 is considered to be highly rapamycin sensitive, whereas mTORC2 is less sensitive, depending on the cell context^40^. In GBM cells, rapamycin can strongly inhibit mTORC1 in the nanomolar range, but mTORC2 can remain insensitive to it, even at relatively high doses^41^. Notably, rapamycin and its analogs may have limited activity against some mTORC1 effectors, including the limited capability to suppress 4E-BP1 phosphorylation in GBM cells^41^. Therefore, the failure of rapamycin (sirolimus) and its analog CCI-779 (temsirolimus)^42^^,^^43^ to suppress mTORC2 signaling, as well as 4E-BP1 phosphorylation in GBM patients, may contribute to clinical resistance.

## mTORC2 reprograms metabolism

**Figure 1:**
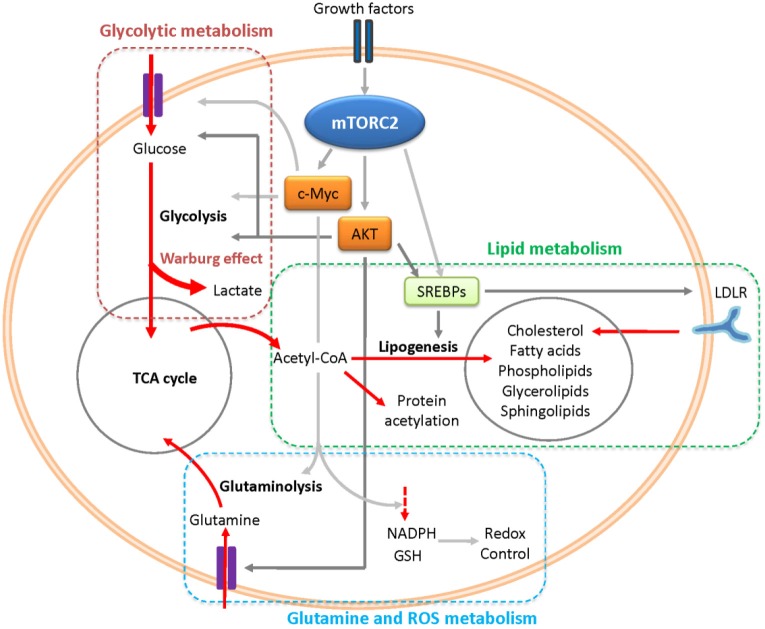
mTORC2 signaling controls metabolic reprogramming in GBM. mTORC2 reprograms the glycolytic metabolism, lipid metabolism, glutamine, and ROS metabolism mainly through AKT and c-Myc. AKT and c-Myc promote glucose uptake, glycolysis, and Warburg effect to generate sufficient ATP and macromolecules for rapid tumor growth. mTORC2 stimulates SREBP cleavage in an AKT-dependent and AKT-independent manner to promote lipogenesis and cholesterol uptake, providing different lipids for the synthesis of membrane and signal molecules. mTORC2 also regulates glutamine uptake and glutaminolysis by activating AKT and c-Myc. The production of NADPH and GSH are increased by mTORC2 in a c-Myc-dependent manner to control cellular redox status. mTOR, mechanistic target of rapamycin; GBM, glioblastoma; SREBP, sterol regulatory binding proteins; NADPH, nicotinamide adenine dinucleotide phosphate; GSH, glutathione.

Metabolic reprogramming is a central hallmark of cancer^44^^,^^45^. Cancer cells need to reprogram their core cellular metabolism to generate sufficient ATP, adequate macromolecules, and appropriate cellular redox status for rapid cell growth, proliferation, and survival^46^. Recent studies suggest that mTORC2 has a central function in metabolic reprogramming, thus contributing to GBM growth and drug resistance. mTORC2 appears to control the metabolism of cancer cells in at least three ways: modulating nutrition (glucose, lipid, amino acid) import, regulating the activity or expression of specific metabolic enzymes, and rewiring of metabolic networks (**Figure 1**).

### Glycolytic metabolism

Cancer cells increase glucose uptake to meet the increased energetic and biosynthetic demands imposed by rapid tumor growth. Even in the presence of sufficient oxygen to support oxidative phosphorylation, tumor cells convert the majority of glucose into lactate. This biochemical adaptation, called “the Warburg effect”, is considered the classic metabolic phenotype of cancer, although it can also be utilized by non-neoplastic, rapidly proliferating cells^47^. The Warburg effect enables the rapidly proliferating cells to utilize the glucose-derived carbons for lipid, ribose, glycerol, serine, and glycine synthesis. Thus, such effect is an efficient method by which cells use glucose for anabolic metabolism while still obtaining sufficient ATP. However, the cells need to take up more glucose for such high cost demands because only two molecules of ATP are yielded per molecule of glucose. Recent studies also demonstrate that mTORC2 serves a central function in the Warburg effect.

First, mTORC2 regulates glycolytic metabolism through its activation of AKT. This complex directly phosphorylates AKT on Ser473 to ensure its maximal activity, thus stimulating the expression of the glucose transporter GLUT4 and activating two key glycolytic enzymes, hexokinase 2 (HK2) and phosphofructokinase-1 (PFK-1); such condition increases glucose uptake and glycolysis^48^^-^^50^. Consistent with this notion, hepatic Rictor knockout mice showed constitutive gluconeogenesis and impaired glycolysis attributed to the loss of AKT Ser473 phosphorylation and reduced glucokinase, which suggests a function of mTORC2 in glycolytic metabolism in normal and cancer cells^51^.

Second, in GBM, mTORC2 promotes the Warburg effect, independent of AKT, by regulating c-Myc levels^52^^,^^53^. A signaling cascade whereby mTORC2 controls the cellular level of c-Myc by inactivating the acetylation of FoxO1 and FoxO3 has recently been identified. The inactivation of FoxOs releases c-Myc from a suppressive miR-34c-dependent network, which targets the 3´-UTR of c-Myc mRNA and inhibits its translation^52^^,^^53^. c-Myc consequently increases the expression of the key regulatory genes controlling glucose transport and glycolysis. These genes include *GLUT1*, *HK2*, pyruvate kinase M2 isoform (PKM2), lactate dehydrogenase A (*LDHA*), and pyruvate dehydrogenase kinase isozyme 1 (*PDK1*), which inhibits pyruvate dehydrogenase (PDH). Thus, c-Myc controls the expression of a repertoire of genes promoting the Warburg effect downstream of mTORC2^52^^,^^53^.

Remarkably, mTORC1 and mTORC2 have been shown to converge on c-Myc through interlacing and complementary pathways. In EGFRvIII-expressing GBMs, mTORC1 functionally regulates the oncogenic activity of c-Myc by inducing the hnRNA1-dependent alternative splicing of the c-Myc-interacting protein, Delta Max^54^. This dual regulation of c-Myc through AKT-mTORC1 and mTORC2 has significant implications on targeted therapy resistance in GBM, as will be discussed below.

Third, mTORC2 potentially suppresses tumor gluconeogenesis by inhibiting the transcriptional level of gluconeogenic genes, thus providing more carbons for the synthesis of macromolecules by cancer cells^52^.

### Lipid metabolism

Along with the reprogramming of glycolytic metabolism, altering the lipid metabolism is increasingly being recognized as another indicator of cancer cells. Tumor cells require abundant amounts of different lipids (including fatty acids, phospholipids, cholesterol, glycerolipids, and sphingolipids) for the synthesis of membranes and signaling molecules. Although mTORC2 is a key regulator in lipogenesis, lipolysis, and adipogenesis in normal cells^51^^,^^55^^-^^60^, its functions in cancer lipid metabolism have not been well established.

Cancer cells are believed to increase *de novo* lipid synthesis and lipid uptake to meet the demands of the rapid proliferation and cell growth^61^^,^^62^. Sterol regulatory binding proteins (SREBPs) are the master transcriptional regulators of lipid uptake and *de novo* lipid synthesis^63^. After being transported from the ER to the Golgi, SREBPs process a series of cleavages on the Golgi membrane, thereafter releasing the helix-loop-helix domain of SREBPs to the nucleus. mTORC1 regulates SREBP function through several mediators, including S6K1 and lipin-1^55^. In GBM, SREBP1 cleavage is activated by the mutant EGFR signaling but is insensitive to rapamycin^64^, which suggests an emerging key function of mTORC2, or is rapamycin-resistant, despite such mTORC1-dependent mechanisms as 4E-BP1 signaling in GBM lipid metabolism reprogramming.

mTORC2 appears to mediate SREBP1 cleavage through AKT-dependent or AKT-independent pathways^51^^,^^56^^,^^64^, thus activating the transcription of key genes in fatty acid and cholesterol *de novo* synthesis. These key genes include acetyl-CoA carboxylase (*ACC*), fatty acid synthase (*FASN*), and acyl-CoA synthetases (*ACS*). mTORC2 also up regulates cholesterol uptake by increasing the expression of LDLR through SREBP1^53^.

Various lipid compositions may confer specific mechanical properties on membranes, as well as necessary oncogenic signals for tumor growth and survival^62^^,^^65^. A recent lipidomic analysis combined with a systematic RNAi screening of lipid biosynthetic enzymes in HeLa cells showed that 11 lipids with specific chemical structures that accumulate in dividing cells are required for cell division^66^. Cancer cells may actively regulate their cellular lipid composition in a similar manner. In yeast, TORC2 senses cellular sphingolipid levels and controls sphingolipid synthesis by activating serine: palmitoyl-coenzyme A transferase (SPT) through the yeast SGK1 homologue Ypk1^67^, which suggests a function of mTORC2 in controlling membrane dynamics. Yeast TORC2 also controls ceramide biosynthesis by regulating the activity of ceramide synthase through Ypk2, the yeast homologue of SGK1^68^.

The mRNA level of lysophospholipase (lysoPLD), which cleaves lysophospholipids to lysophosphatidic acid (LPA), is significantly increased in glioma cells^69^. Cellular phosphatidic acid has been shown to be an activator of mTORC1 and mTORC2^19^. Whether mTORC2 contributes to the control of a specific lipid composition and how mTORC2 signaling regulates the phospholipid metabolism in cancer cells remain unclear.

Acetyl-CoA, the substrate for *de novo* fatty acid synthesis, as well as histone acetylation, serves an important function in the epigenetic regulation of cancer cells. This substrate may be regulated by mTORC2 signaling through the control of ATP citrate lyase (ACLY) expression, which catalyzes the first step of fatty acid synthesis that converts citrate to acetyl-CoA^70^. The genetic depletion of rictor in the My5 precursor cells gives rise to myocytes, brown adipose tissue, and a subset of white adipose tissue, thus significantly reducing the level of ACLY in brown fat adipose tissue and protecting against the development of obesity in mice fed with a high-fat diet under thermoneutral conditions^71^. In cancer cells, increased glycolysis provides sufficient citrate from the TCA cycle for acetyl-CoA synthesis through ACLY^72^. Thus, mTORC2 may potentially regulate histone and protein acetylation in GBM by controlling ACLY levels.

### Glutamine and ROS metabolism

mTORC2 controls glutamine uptake by regulating the cell surface amino acid transporters, SNAT2 and LAT1, through AKT and SGK activation^73^^,^^74^. This complex also up regulates glutaminase levels to promote glutaminolysis through c-Myc^52^. The high levels of reactive oxygen species (ROS) derived from rapid proliferation can damage cancer cells. Reduced nicotinamide adenine dinucleotide phosphate (NADPH) and glutathione (GSH), two of the most abundant antioxidants, are responsible for controlling the increase in cellular ROS levels to maintain cellular redox status in cancer cells^46^. mTORC2 regulates the production of NADPH and GSH in a c-Myc-dependent manner by promoting the expression of enzymes in NADPH and GSH synthesis^46^^,^^52^.

## mTORC2 is a central regulator of drug resistance pathways

mTORC2 can potentially contribute to cancer drug resistance via several pathways. First, mTORC2 may contribute to chemotherapy resistance directly through its activation of AKT. Second, mTORC2 can promote resistance through SGK-1-dependent and NF-κB-dependent signaling^23^^,^^38^. Interestingly, the activation of the NF-κB pathway in this case is not AKT-dependent, though AKT has been shown to regulate NF-κB signaling in various other circumstances^75^^,^^76^. This finding suggests that GBMs have developed additional chemotherapy resistance mechanisms because AKT inhibition alone will be insufficient to sensitize tumors to chemotherapy.

Several studies suggest a link between dysregulated metabolism and cancer drug resistance. As an example, LDHA, which regulates aerobic glycolysis, has been associated with taxol and trastuzumab resistance; silencing LDHA is capable of sensitizing cancer cells to these drugs^77^^,^^78^. A *de novo* lipogenesis gene FASN has also been recognized as a drug resistance factor in breast cancer cells^79^. However, the exact mechanism by which reprogrammed metabolism regulates drug resistance remains unknown. Given that mTOR is a central regulator of cellular metabolism, the notion that oncogenic activation of mTOR signaling promotes drug resistance through metabolic reprograming remains an open question.

c-Myc, a critical metabolic regulator^80^, is controlled by a dual-pronged mechanism downstream of growth factor signaling in GBM cells^52^^,^^53^, which may have significant implications for the resistance to PI3K, AKT, and mTOR-targeted therapies. c-Myc levels are regulated through FoxO1 and FoxO3 by two different types of post translational modifications. As a result of AKT and/or mTORC1-dependent phosphorylation, FoxO phosphorylation results in exclusion from the nucleus and de-repression of c-Myc from miR-145-dependent suppression^81^. mTORC2-dependent FoxO acetylation de-represses c-Myc from miR-34c^52^. Thus, this dual-pronged regulation may cause the insufficiency of PI3K, AKT, and/or mTORC1 inhibitors, including combinations that are being tested in early to mid-phase clinical trials, to suppress the cancer metabolic reprogramming through c-Myc. This condition will result in clinical resistance.

mTORC2 inhibition can also be required to mitigate c-Myc expression and metabolic reprogramming to achieve clinical remission. The activity of mTORC2 appears to be significantly more difficult to suppress than that of mTORC1, despite having ATP-competitive mTOR kinase inhibitors. This observation suggests that inhibitors with enhanced activity against mTORC2, including allosteric inhibitors, may be needed.

## Targeting mTOR signaling for cancer therapy

Rapamycin and its analogues (rapalogues) that allosterically inhibit mTORC1 exhibit limited activity against mTORC2, although some inhibitory effect has been documented in certain cell types with prolonged rapamycin treatment^40^. This condition highlights the need for a strategy to interrupt mTORC2 specific protein-protein interactions. In contrast to allosteric inhibitors, the ATP-competitive mTOR inhibitors that directly inhibit mTOR kinase activity by competing with ATP for binding to the kinase domain of mTOR have the potential to inhibit both mTORC1 and mTORC2. In GBM, the mTOR kinase inhibitors CC214-1 and CC214-2 inhibit rapamycin-resistant mTORC1 and mTORC2 signaling, thus blocking protein translation, cell proliferation, and tumor growth. Interestingly, EGFRvIII expression and PTEN loss sensitized tumor cells to CC214 compounds, thereby implying that GBM cells with higher levels of PI3K signaling may be more sensitive to the interruption of mTOR signaling^41^.

The similarity of the kinase domains between mTOR and PI3Ks and the importance of PI3K signaling dependent of mTOR can cause ATP-competitive PI3K/mTOR kinase inhibitors to serve a key function in targeted cancer treatment.

Pharmacokinetic failure appears to be a major cause of resistance to mTOR and dual PI3K/mTOR kinase inhibitors in patients. Considerable effort will be needed to improve the pharmacokinetic properties of these drugs *in vivo* and to optimize the therapeutic dosing regimens to enable patients to overcome the resistance caused by insufficient drug exposure and to prevent the failure to inhibit mTOR signaling sufficiently. Doses may be sufficient to block mTOR signaling in circulating white blood cells but are inadequate to inhibit intratumoral mTOR signaling in patients.

Cloughesy *et al*.^82^ have reported that the inhibition of GBM cell proliferation is correlated with the magnitude of mTOR inhibition in a Phase I rapamycin trial of GBM patients. However, even the concentrations of rapamycin in most GBM tissues were above the level known to confer anti-proliferative activity in PTEN-null cell lines *in vitro* (typically 1 nM), not every patient showed corresponding biological responses (determined by Ki-67), partially because of inadequate mTOR signaling inhibition (determined by S6 phosphorylation). One of the possible mechanisms is that rapamycin can be sequestered in red blood cells^83^, thus making the actual concentration in tumor cells inadequate for target inhibition. Rapamycin is not the only targeted therapy to fail in sufficiently suppressing intratumoral drug targets in patients; such failure has also been noted for the EGFR inhibitors erlotinib and lapatinib^84^.

To overcome such intrinsic resistance, the pharmacokinetic properties of the drugs should be improved, and the dosing regimens should be optimized to achieve higher concentrations in tumor cells. Das Thakur *et al*.^85^ demonstrated that the continuous dosing of vemurafenib in BRAF-mutated melanoma mice over an extended period results in the emergence of resistant tumors. By contrast, intermittent dosing can re-sensitize tumors to vemurafenib *in vivo*^85^. An attractive question worthy of further exploration is whether discontinuous treatment with higher doses of targeted drugs will achieve more sufficient inhibition and yield better clinical outcomes for GBM patients.

## Conclusion

In this review, we emphasized the important functions of mTORC2 in GBM, with a focus on its contributions to metabolic reprogramming and drug resistance. mTORC2 appears to be essential in GBM pathogenesis and is thus worth serious consideration as a drug target. However, the knowledge about mTORC2 signaling remains limited. Many questions still remain on how exactly is mTORC2 being regulated and on what is the best method to suppress it. Other questions include those on the key partners in the metabolic reprogramming of GBM and the significance of the function of mTORC2 in different types of cancer. Future mechanistic studies are required to further elucidate the function of mTORC2 in cellular growth and metabolism, given the emergence of mTORC2 as a compelling clinical drug target along with mTORC1.
